# Pandemic Influenza Virus 2009 H1N1 and Adenovirus in a High Risk Population of Young Adults: Epidemiology, Comparison of Clinical Presentations, and Coinfection

**DOI:** 10.1371/journal.pone.0085094

**Published:** 2014-01-08

**Authors:** Heather C. Yun, William H. Fugate, Clinton K. Murray, Thomas L. Cropper, Lisa Lott, J. Matthew McDonald

**Affiliations:** 1 San Antonio Military Medical Center, Joint Base San Antonio Fort Sam Houston, Texas, United States of America; 2 Center for Advanced Molecular Detection, 59th MDW/ST, Joint Base San Antonio-Lackland, Texas, United States of America; 3 Trainee Health Surveillance, Joint Base San Antonio-Lackland, Texas, United States of America; 4 Uniformed Services University of the Health Sciences, Bethesda, Maryland, United States of America; Naval Medical Research Unit 6, United States of America

## Abstract

**Background:**

In 2009, pandemic H1N1 influenza virus (2009 H1N1) emerged worldwide, causing morbidity and mortality that disproportionately affected young adults. Upper respiratory infection (URI), largely due to adenovirus, is an endemic cause of morbidity in military training. Whether clinical presentations differ or excess morbidity results from coinfection is unclear.

**Methods:**

The Center for Advanced Molecular Detection evaluates epidemiology and rapid diagnostics of respiratory pathogens in trainees with URI. From May 1, 2009, to November 30, 2009, demographic, clinical, and PCR data from throat and nasal specimens for adenovirus and 2009 H1N1 were prospectively collected.

**Results:**

375 trainees with URI enrolled and were tested for both adenovirus and 2009 H1N1 by PCR (median age 20; 89% male). Adenovirus PCR was positive in 72% (96% serotype E-4) and 2009 H1N1 in 20%. Males were more likely to have adenovirus and females more likely to have 2009 H1N1 (p  =  0.047). Subjects with 2009 H1N1 presented an average of 1 week earlier in training, had shorter illness duration before enrollment, less sore throat, diarrhea, and fewer abnormal findings on throat exam. Coryza and cough were more common with 2009 H1N1 compared to adenovirus. Subjects with 2009 H1N1 were less likely to have adenovirus than those without, despite persistently high frequencies of adenovirus detections during peak 2009 H1N1 weeks (15% vs. 83%, p < 0.01). Coinfection with adenovirus and 2009 H1N1 was rare (4%). Rates of hospitalization and pneumonia did not differ between the adenovirus, 2009 H1N1, or coinfected groups.

**Conclusion:**

Military trainees with 2009 H1N1 vs. adenovirus have differing clinical presentations, and males are more likely to have adenovirus. Despite high frequencies of adenovirus infection, coinfection with adenovirus and 2009 H1N1 is rare and apparently does not result in increased morbidity.

## Introduction

Non-influenza related upper respiratory infections (URI) are universally experienced illnesses that, despite their typically self-limited nature, lead to billions of dollars of lost income, and predispose to serious illnesses including pneumonia.[1] When influenza is responsible, pandemics can result and cause millions of deaths. In 2009, a novel H1N1 influenza virus (2009 H1N1) emerged and rapidly spread worldwide, causing excess mortality in children and young adults. Although the global estimate of deaths has been lower than seen in several previous pandemics, the number of life years lost is estimated to be five times higher than those lost to seasonal H1N1 viruses and comparable to the number lost during the 1968 pandemic.[2], [3] Military trainees, along with other groups of crowded, stressed individuals, are disproportionately affected by respiratory illnesses due to a variety of pathogens. With the exception of the prior adenovirus vaccine era from 1980–1996, adenoviruses have historically been the most common causes of febrile URI in this population, and have also led to serious illness and fatalities.[4]–[7] In one large study of transmission dynamics of adenovirus in a military training setting, approximately one-third of incoming trainees were already immune, one-third developed a febrile URI due to adenovirus, and the remainder seroconverted with subclinical or asymptomatic infection.[8] Large influenza outbreaks are less common, given the universal immunization of basic trainees and routine use of ring antiviral chemoprophylaxis in training units with known influenza cases, if cases occur within the first two weeks after immunization.[9], [10] However, in 2009, type-specific influenza vaccine was not widely available until well into the full wave of illness.[11] With large numbers of concurrently circulating respiratory pathogens occurring year round in this diverse group of individuals, coming from a variety of geographic locations and backgrounds, and living in close contact for months, coinfection with multiple organisms would be expected to be a regular occurrence. However, whether coinfection contributes to differing clinical presentations or outcomes in this young, healthy adult population is unknown. While coinfections with viral pathogens including 2009 H1N1 have been described in patients with respiratory infections, few prospective studies have related these to clinical presentation and outcomes in adults since molecular diagnostics became available, and none in the setting of high background rates of adenovirus.[12]–[17]

We sought to describe the epidemiology of 2009 H1N1 and adenovirus in a basic training population, and to correlate differences in clinical presentations and outcomes with each respective pathogen and in coinfections.

## Methods

### Setting

Joint Base San Antonio-Lackland is the only Air Force location for basic military training with approximately 43,000 recruits per year, 6,000–7,000 recruits training at any given time, and a training period lasting 8.5 weeks. Basic military trainees (BMTs) are assigned to training units called “flights” of 50–60 individuals, with whom they train and reside in bay dormitories; tobacco product use is not allowed. Ill trainees present for care at an outpatient clinic; if they are febrile with a respiratory illness they are then cohorted to a “fever flight” where they recover until they are afebrile and able to return to training. Trainees who require hospitalization are admitted to the tertiary care hospital on base. Trainees routinely receive chemoprophylaxis against *Streptococcus pyogenes* during their first week of training; this consists of benzathine penicillin or azithromycin for penicillin allergic recruits. Immunizations against meningococcus, hepatitis A and B, and measles, mumps and rubella are also administered during the first week of training. Trivalent seasonal influenza vaccine was administered during the first week of training throughout the study period, but 2009 H1N1 vaccine was not available until December 1, 2009. During the study period, oseltamivir was routinely used for treatment of ill trainees with confirmed 2009 H1N1 infection. Oseltamivir was also routinely used for chemoprophylaxis of well trainees in close contact with a confirmed case.

### Study design

The Center for Advanced Molecular Detection (59^th^ Medical Wing/Science and Technology, Air Education and Training Command) was established in 2003 for prospective evaluation of epidemiology and novel technologies to rapidly detect respiratory pathogens in trainees with URI. Subjects were approached for enrollment at the point of care for their URI, and met inclusion criteria if they were BMTs 17 years of age or older and had any symptom of upper respiratory tract infection or pneumonia. Demographic data, including age, race, gender, week of training, city/state of previous residence, and smoking history, were recorded. Additionally, a symptom questionnaire (including respiratory and gastrointestinal symptoms), perceived stress level on a 10-point Likert scale, and clinical signs, including vital signs, height and weight, physical exam findings, and physician diagnosis were recorded, as was the ward of hospital admission (intensive care unit vs. ward) where applicable. For this substudy, cases were included if they enrolled in the study and were tested for both adenovirus (using study methodology) and 2009 H1N1 (as part of clinical care). Duplicate cases (for numerous presentations for URI in the same subject) were excluded; all cases represent unique subjects.

### Clinical laboratory data

Respiratory viral culture data (Wilford Hall Medical Center) and 2009 H1N1 influenza virus PCR data (United States Air Force School of Aerospace Medicine reference laboratory) obtained during clinical care were prospectively collected from May 1, 2009, to November 30, 2009. Both respiratory viral culture and 2009 H1N1 PCR were performed on predominantly nasal wash specimens as part of routine clinical care. Respiratory viral culture was performed using standard methods and 2009 H1N1 PCR using the CDC protocol of real-time RTPCR for influenza A (H1N1) (World Health Organization Collaboration Center for Influenza at the Centers for Disease Control and Prevention, Atlanta, GA, USA).[18]

### Nucleic acid extraction

Nasal wash and throat swabs for adenovirus PCR were collected in parallel in approximately 3 ml of saline and viral transport medium, respectively. Total nucleic acid was extracted from 400µl of each sample using the MagNA Pure Compact Nucleic Acid Isolation Kit I (Roche Diagnostics, Mannheim, Germany, MagNA Pure Kit 03 730 964 001) with the MagNA Pure Compact instrument (Roche Applied Science Mannheim, Germany).

### Real-time adenovirus PCR and data interpretation

Primers and probes used have been reported for adenovirus by Heim et al.[19] All qPCR was conducted using Applied Biosystems 7900 and 7500 real-time PCR instruments (Applied Biosystems, CA).

For adenovirus testing, cycling was conducted with 500 nM concentrations of both forward and reverse primers and 300 nM concentration of probe. Reaction conditions included an initial 10 min denaturation at 95°C, followed by 45 cycles of 95°C for 15 sec and 60°C for 1 min. A specimen was considered positive if its cycle of threshold (Ct value) was equal to or less than 40, as described previously.[19], [20].

### Protection of human subjects/Ethics Statement

All subjects provided written, voluntary informed consent in the presence of an ombudsman. The study was approved by Wilford Hall Medical Center/Brooke Army Medical Center Institutional Review Board (IRB). Gender and ethnicity were self-reported. Per Department of Defense (DoD) Directive 3216.02, for purposes of legal capacity to participate in DoD-conducted or -supported research involving human subjects, all active duty service members in a federal duty status are considered to be adults. The participation of such members is not subject to requirements regarding research involving children or minors. When service members are under 18 years of age, students at service academies, or trainees, the IRB shall carefully consider the recruitment process and the necessity to include such members as human subjects. The Wilford Hall Medical Center/Brooke Army Medical Center IRB carefully considered the BMT recruitment process for the study and ruled that consenting BMTs 17 years of age or older who presented to the health clinics could be enrolled.

### Statistical analyses

Data were entered in duplicate for quality control. Analysis was performed using existing software (SPSS, version 19.0; SPSS). Continuous variables were analyzed by Student’s t-test or Mann-Whitney U test for parametric and nonparametric data, respectively. Categorical variables were evaluated by chi-squared, Fisher’s exact test, or Spearman correlation. Multiple nonparametric groups of continuous variables were analyzed by Kruskal-Wallis testing. All p-values are two-tailed and statistical significance at p <0.05.

## Results

**Figure 1:**
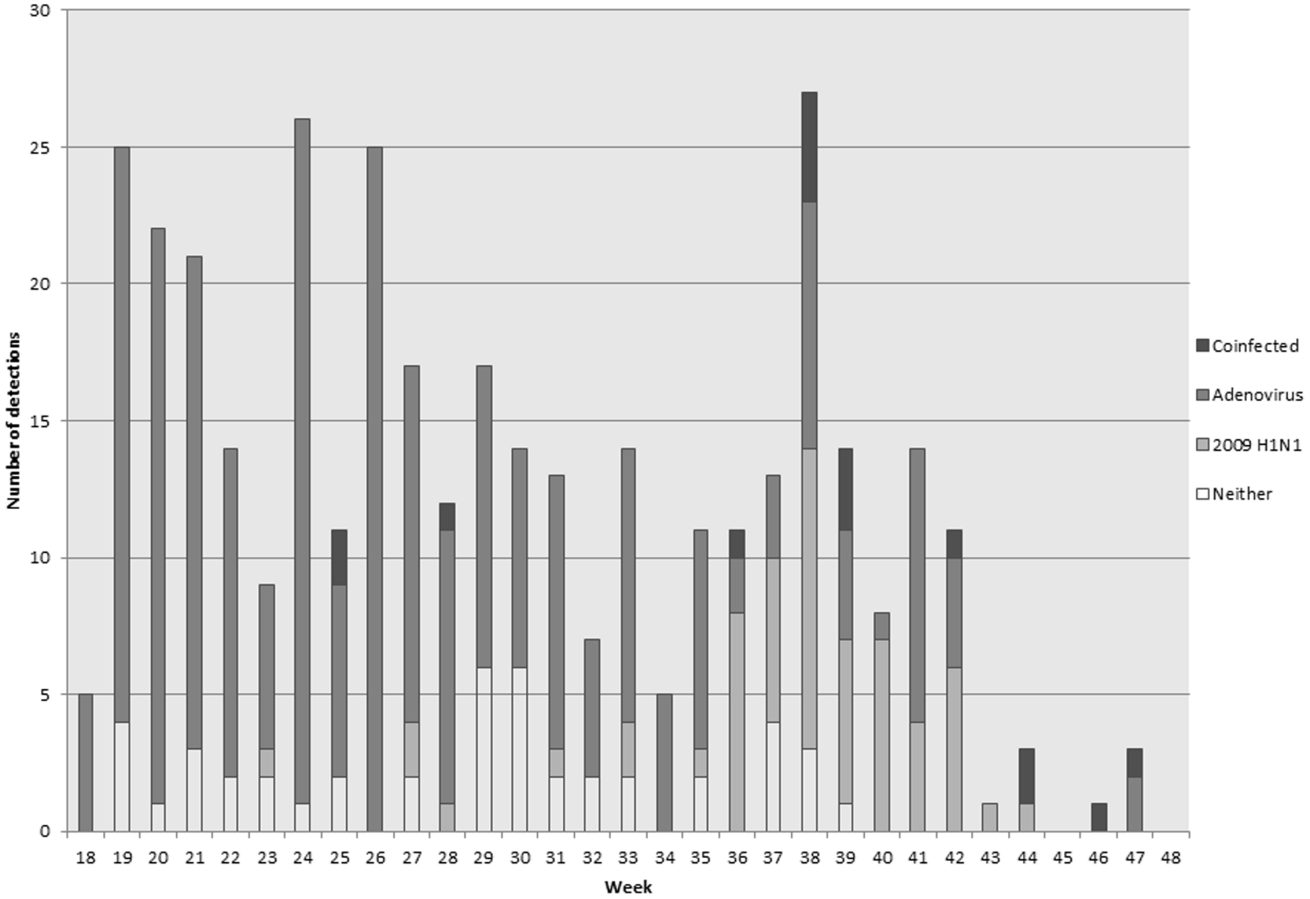
Number of detections of adenovirus, 2009 H1N1 influenza virus, and coinfected from May 1, 2009- November 30, 2009.

From May 1, 2009, to November 30, 2009, 375 subjects with URI were enrolled and tested for both adenovirus and 2009 H1N1. The baseline characteristics of the study population are shown in Table 1. Men constituted 89% of the study population. The median age was 20 years (IQR 19–22), and 69% were white. Adenovirus PCR was positive in 72% of subjects and 20% were positive for 2009 H1N1 by PCR; 4% were coinfected. Adenovirus E-4 was the most commonly recovered serotype, accounting for 96% of all adenovirus detections; serotypes B-7 and B-14 were infrequently detected. Specific pathogen recovery data is presented in Table 2, and seasonal variation presented in Figure 1.

**Table 1:** Demographic information of trainees presenting with respiratory illness.

	Total population	
Gender	375	
Male		89.3%
Female		10.7%
Age	374	20 (IQR 19–22)
Race-Ethnicity	358	
White		69.3%
Black		15.4%
Hispanic		10.3%
Asian		2.5%
Native American		0.3%
Other/Multiple		2.2%
Week of Training	371	6 (IQR, 4–7)
Body Mass Index (kg/m^2^)	374	23.6 (IQR, 21.7–25.1)
Perceived Stress Level*	373	4 (IQR 3–5)
History of smoking	375	13.9%

IQR, interquartile range.

Ten point Likert scale where 10 represents maximal subjective stress and 0 is no stress at all.

**Table 2:** Pathogens recovered, by specimen source.

	N tested	N positive
Viral culture	375	
Adenovirus (Ad)		224 (59.7%)
Influenza virus		73 (19.5%)
Parainfluenza type 3		1 (0.3%)
2009 H1N1 PCR	375	74 (19.7%)
Ad Pan PCR		
Nasal Wash	365	242 (66.3%)
Throat Swab	367	254 (69.2%)
Ad B-14 PCR		
Nasal Wash	373	3 (0.8%)
Throat Swab	375	4 (1.1%)
Ad E-4 PCR		
Nasal Wash	373	254 (68.1%)
Throat Swab	375	260 (69.3%)
Ad B-7 PCR		
Nasal Wash	63	1 (1.6%)
Throat Swab	65	1 (1.5%)
Any Ad PCR	375	271 (72.3%)
Coinfected: Any Ad PCR, 2009 H1N1 PCR	375	16 (4.3%)

During the peak of 2009 H1N1 activity among trainees, weeks 33–42, out of 130 subjects, adenovirus was found alone in 58, 2009 H1N1 alone in 51, and 9 were coinfected; neither virus was recovered from 12 subjects. During that time frame, there was a negative association between 2009 H1N1 and adenovirus (p<0.01). Subjects with 2009 H1N1 were much less likely to be adenovirus positive than those without 2009 H1N1 (15% vs. 83%). Subjects with adenovirus were much less likely to have 2009 H1N1 than those without adenovirus (13% vs. 81%).

A comparison of demographic information in subjects infected with 2009 H1N1 vs. adenovirus is presented in Table 3. Significant findings include gender disparities of 2009 H1N1 and adenovirus infection, with a larger proportion of males presenting with adenovirus (p  =  0.047). Including subjects with coinfections, adenovirus was detected in 60% of female subjects vs. 74% of male subjects, and 2009 H1N1 in 30% of female subjects vs. 19% of male subjects. 2009 H1N1 infected subjects also presented one week earlier in training. However, after exclusion of cases diagnosed in the first week of training, the distributions of the epidemic curves were similar and both peaked in the fifth week (data not shown). Adenovirus detections peaked earlier in the year compared to 2009 H1N1 infections.

**Table 3:** Demographic information of trainees presenting with upper respiratory infection and infected with adenovirus (Ad) or 2009 H1N1 alone (n  =  313).

	Ad +(n = 255)	2009 H1N1 +(n = 58)	p-value
Gender			0.047*
Male	234 (91.8%)	49 (84.5%)	
Female	21 (8.2%)	9 (15.5%)	
Age (IQR)	20 (19–21)	20 (18–22)	0.54
Race-Ethnicity			0.34*
White	170 (68.8%)	40 (74.1%)	
Black	40 (16.2%)	4 (7.4%)	
Hispanic	25 (10.1%)	7 (13.0%)	
Asian	8 (3.2%)	1 (1.9%)	
Other/Multiple	4 (1.6%)	2 (3.7%)	
Week of Training	6 (5–7)	5 (3–5.25)	<0.01
Body mass index (kg/m^2^)	23.7 (21.9–25.1)	23.6 (21.3–25.3)	0.28
Perceived Stress Level**	4 (3–5)	5 (3–6)	0.43
Smoking history	34 (13.1%)	8 (13.3%)	1.0
Week of year (IQR)	26 (21–31)	38 (36–40)	<0.01

IQR, interquartile range.

Spearman correlation.

Ten point Likert scale where 10 represents maximal subjective stress and 0 is no stress at all.

The most commonly reported symptoms were fever (100%), cough (90%), headache (88%), and sore throat (87%), and the median duration of symptoms prior to enrollment was 3 days (Table 4). Six patients were diagnosed with pneumonia, and three were admitted to the hospital, all to regular internal medicine wards. Clinical differences, presented in Table 4, included a shorter duration of illness prior to presentation for clinical care in the 2009 H1N1 infected group compared to the adenovirus group (2 days vs. 3 days, p < 0.01), increased proportion of subjects complaining of coryza and cough in the 2009 H1N1 group, and increased predominance of sore throat and diarrhea in the adenovirus group. Physical exam revealed increased abnormal findings on throat exam in the adenovirus group. There were no differences in pneumonia or hospitalization rates between the two groups, but both of these events were rare.

**Table 4:** Clinical characteristics of total study population, and comparison of clinical variables: Adenovirus (Ad) vs. 2009 H1N1, and coinfection vs. Ad alone.

	Total study population (n = 375)	Ad +(n = 255)	2009 H1N1 +(n = 58)	p-value	Ad+/2009 H1N1+(n = 16)α	p-value
Symptoms						
Subjective fever	373 (99.5%)	253 (99.2%)	58 (100%)	1.0	16 (100%)	1.0
Cough	336 (89.6%)	226 (88.6%)	56 (96.6%)	0.01	15 (93.8%)	1.0
Sore throat	326 (86.9%)	236 (92.5%)	43 (74.1%)	<0.01	11 (68.8%)	<0.01
Sinus congestion	301 (80.3%)	206 (80.8%)	44 (75.9%)	0.34	13 (81.3%)	0.26
Myalgia	295 (78.7%)	196 (76.9%)	47 (81.0%)	0.49	15 (93.8%)	0.21
Coryza	241 (64.3%)	155 (60.8%)	45 (77.6%)	0.02	12 (75.0%)	0.12
Malaise	198 (52.8%)	132 (51.8%)	35 (60.3%)	0.25	8 (50.0%)	0.88
Vomiting	45 (12.0%)	32 (12.5%)	6 (10.3%)	0.64	3 (18.8%)	0.44
Diarrhea	21 (5.6%)	20 (7.8%)	0	0.03	0	0.62
Duration of symptoms (days; IQR*)	3 (2–4)	3 (2–5)	2 (2–3)	<0.01	2.5 (2–3)	0.09
Vital Signs**						
Heart Rate	94.1 (14.1)	93 (15)	97 (12)	0.08	95 (10)	0.71
Respiratory Rate	17.0 (1.8)	17 (2)	17 (1)	0.92	17 (2)	0.37
Systolic BP***	119.6 (9.3)	120 (9)	119 (11)	0.67	120 (11)	0.92
Diastolic BP***	71.9 (7.5)	72 (7)	71 (9)	0.13	71 (3)	0.61
Oral temperature (^0^F)	101.5 (0.9)	101.5 (0.8)	101.7 (1.0)	0.07	101.9 (0.9)	0.03
Physical exam						
Pharyngitis	283 (75.5%)	203 (79.6%)	38 (65.5%)	<0.01	9 (56.2%)	<0.01
Exudativepharyngitis	22 (5.9%)	21 (8.2%)	1 (1.7%)	0.045	0	0.24
Lymphadenopathy	189 (50.4%)	131 (51.4%)	32 (55.2%)	0.78	5 (31.3%)	0.08
Tonsillitis	64 (17.1%)	53 (20.8%)	2 (3.4%)	<0.01	0	0.02
Abnormal lung exam	11 (2.9%)	7 (2.7%)	2 (3.4%)	0.30	0	0.63
Pneumonia	6 (1.6%)	4 (1.6%)	1 (1.7%)	1.00	0	0.78
Hospitalized	3 (0.8%)	2 (0.8%)	0	1.00	0	1.00

median, interquartile range.

mean, standard deviation.

BP  =  blood pressure (mmHg).

compared to Ad alone.

Clinical data in subjects infected with adenovirus, with or without concomitant 2009 H1N1, are also presented in Table 4. Sore throat was less common in the coinfection group compared to adenovirus alone (71% vs. 94%, p  =  0.01), and abnormalities on throat exam were less common in the coinfection group compared to adenovirus alone. Oral temperature on presentation was higher for the coinfected group than for those infected with adenovirus alone (101.9^0^F vs. 101.5^0^F, p  =  0.03). There were no hospitalizations or diagnoses of pneumonia in the coinfection group; this was not significantly different than the rates seen in the adenovirus group.

## Discussion

This large, prospective cohort study describes otherwise healthy, young adult patients presenting with acute febrile respiratory illness during basic military training and evaluated, through PCR amplification of respiratory specimens, for 2009 H1N1 infection, adenovirus, or both. These data illustrate the scarcity of coinfections with 2009 H1N1 and adenovirus, despite high endemic frequencies of adenovirus in this population during peak 2009 H1N1 months. This study also represents the largest clinical evaluation of adenovirus and 2009 H1N1 coinfected patients to date, to our knowledge. In the wake of 2009 H1N1 emergence, a number of studies have investigated the role of coinfection with viral pathogens with 2009 H1N1. One evaluation of non-influenza viruses in influenza-like illness during the 2009 H1N1 epidemic in France demonstrated a 5% incidence of adenovirus; only 1 patient was coinfected with adenovirus and 2009 H1N1, limiting clinical evaluation of this particular combination.[17] Several additional studies have sought to examine whether clinical presentations vary with 2009 H1N1 infection in the presence of other respiratory viruses, with at least one suggesting increased clinical severity among some non-rhinovirus coinfections, but these have included few adenovirus coinfections.[16], [21].

A number of studies have also evaluated whether 2009 H1N1was associated with either negative or positive effects (predominantly in terms of acquisition rather than severity) on other respiratory viruses, and, taken together with reference to adenovirus, the results of these are inconclusive. One study performed in the United Kingdom in 2009–2010 suggested negative associations between 2009 H1N1 and human metapneumovirus as well as rhinovirus, though not adenovirus. However, few adenovirus detections were found compared to our population (∼5%), and most of these were in young children.[22] A South African study of respiratory viruses in hospitalized patients found only six patients with adenovirus and 2009 H1N1 coinfection out of over 8000 subjects enrolled.[23] To our knowledge, negative associations between adenovirus and influenza A virus (either seasonal or 2009 H1N1) detection have not been demonstrated. It is also unclear, if there is a negative association between the two viruses, whether adenovirus is protective against influenza infection or vice versa. In this data set, influenza patients presented earlier, although not after excluding those presenting during the first week of training, who likely would have arrived with their infection. The policy of cohorting together all BMTs with fever and URI, regardless of the causative pathogen, would seem to increase the likelihood of coinfection, rather than skewing the data towards the appearance of a negative association. It will also be worthwhile to see whether influenza epidemiology will change since the late 2011 reintroduction of adenovirus serotypes 4 and 7 vaccines in military trainees, or whether issues arise with concurrent administration of both live attenuated influenza and adenovirus vaccines, which could affect current trainee vaccine policies. In the meantime, concerns about cohorting patients that may have either adenovirus or 2009 H1N1 on the basis of syndromic presentation can be alleviated on the relative scarcity of coinfection and on the absence of any evidence of increased illness severity among coinfected subjects.

The differing demographics and clinical presentations of adenovirus vs. 2009 H1N1 infection in this study are also of interest. First, the gender differences seen for each virus are intriguing. Males represented 89% of the study population, enrolled after presenting with respiratory illness. However, males generally represent only 80% of the Air Force basic training population, so the study population was already disproportionately male. Adenovirus has long been suggested to be predominantly an illness of men in this population, and this study is no exception to this trend.[24], [25] 2009 H1N1, like all influenza A viruses, has similar mechanisms of transmission, yet in this study population disproportionately affected females. The reasons for this gender difference are unclear. Both of these infections as captured in this study population would meet the CDC definition for influenza-like-illness (fever plus cough or sore throat), used for surveillance and cohorting purposes, but had differences in presentation of statistical and arguably clinical significance.[26] Adenovirus was consistently more likely to produce signs and symptoms referable to the throat, while 2009 H1N1 produced a predominance of cough, as well as shorter illness duration prior to presentation for care, potentially reflecting more rapid development of uncomfortable symptoms. These findings may have importance for both infection control and empiric therapy.

The strengths of this study include the molecular characterization of respiratory pathogens together with capture of detailed clinical data in a large cohort of otherwise healthy adult patients with few complicating comorbidities, as well as the closed nature of the population, which lends itself to capture of events such as hospital admissions and pneumonia diagnoses. Limitations include the absence of serologic data, or the ability to serially characterize pathogens from the same cohort of individuals, which would provide more granular detail about the time course of developing each infection. Subject enrollment was variable throughout the study period, depending on rates of clinical illness within the training population, as well as availability of study personnel to enroll trainees, and given that 2009 H1N1 influenza virus PCR was done as part of clinical care, there could have been some differences in those who enrolled vs. did not enroll, or those who received 2009 H1N1 testing (and thus were included in this study) and those who did not. Due to this variability in total enrollment, and because 2009 H1N1 testing became infrequent clinically after November 2009, inferences about the impact of H1N1 towards the end of 2009 are limited. However, data from the Naval Health Research Center’s Febrile Respiratory Illness Surveillance Update show a rate of febrile respiratory infection that increased in December of 2009, 82% of which was associated with adenovirus, with no influenza virus detected.[27] Additionally, the use of oseltamivir, both for prophylaxis and for treatment, would have impacted both epidemiology and severity of illness. Finally, without inclusion of asymptomatic or minimally symptomatic individuals, conclusions can only be drawn about the scarcity of coinfections with individuals at the point of presentation to care.

In summary, this epidemiologic survey of young adults in military training presenting with fever and URI demonstrated significant differences in 2009 H1N1 vs. adenovirus in terms of gender predilection and presenting symptoms. In addition, coinfections with 2009 H1N1 and adenovirus were rare despite high endemicity of adenovirus before and during the 2009 H1N1 epidemic, and, beyond a higher temperature on presentation, coinfections were not associated with increased clinical severity compared with adenovirus alone.
